# Vitamin D Status and Bone and Connective Tissue Turnover in Brown Bears (*Ursus arctos*) during Hibernation and the Active State

**DOI:** 10.1371/journal.pone.0021483

**Published:** 2011-06-23

**Authors:** Peter Vestergaard, Ole-Gunnar Støen, Jon E. Swenson, Leif Mosekilde, Lene Heickendorff, Ole Fröbert

**Affiliations:** 1 The Osteoporosis Clinic, Aarhus University Hospital, Aarhus, Denmark; 2 Department of Ecology and Natural Resource Management, Norwegian University of Life Sciences, Ås, Norway; 3 Norwegian Institute for Nature Research, Trondheim, Norway; 4 Department of Endocrinology and Internal Medicine, Aarhus University Hospital THG, Aarhus, Denmark; 5 Department of Clinical Biochemistry, Aarhus University Hospital, Aarhus, Denmark; 6 Department of Cardiology, Örebro University Hospital, Örebro, Sweden; University of Queensland, Australia

## Abstract

**Background:**

Extended physical inactivity causes disuse osteoporosis in humans. In contrast, brown bears (*Ursus arctos*) are highly immobilised for half of the year during hibernation without signs of bone loss and therefore may serve as a model for prevention of osteoporosis.

**Aim:**

To study 25-hydroxy-vitamin D (25OHD) levels and bone turnover markers in brown bears during the hibernating state in winter and during the active state in summer. We measured vitamin D subtypes (D_2_ and D_3_), calcitropic hormones (parathyroid hormone [PTH], 1,25-dihydroxy-vitamin D [1,25(OH)_2_D]) and bone turnover parameters (osteocalcin, ICTP, CTX-I), PTH, serum calcium and PIIINP.

**Material and Methods:**

We drew blood from seven immobilised wild brown bears during hibernation in February and in the same bears while active in June.

**Results:**

Serum 25-hydroxy-cholecalciferol (25OHD_3_) was signifi**c**antly higher in the summer than in the winter (22.8±4.6 vs. 8.8±2.1 nmol/l, two tailed p **-** 2p = 0.02), whereas 25-hydroxy-ergocalciferol (25OHD_2_) was higher in winter (54.2±8.3 vs. 18.7±1.7 nmol/l, 2p<0.01). Total serum calcium and PTH levels did not differ between winter and summer. Activated 1,25(OH)_2_D demonstrated a statistically insignificant trend towards higher summer levels. Osteocalcin levels were higher in summer than winter, whereas other markers of bone turnover (ICTP and CTX-I) were unchanged. Serum PIIINP, which is a marker of connective tissue and to some degree muscle turnover, was significantly higher during summer than during winter.

**Conclusions:**

Dramatic changes were documented in the vitamin D_3_/D_2_ ratio and in markers of bone and connective tissue turnover in brown bears between hibernation and the active state. Because hibernating brown bears do not develop disuse osteoporosis, despite extensive physical inactivity we suggest that they may serve as a model for the prevention of this disease.

## Introduction

Bone mineral density in humans is tightly regulated by loading and physical activity [1]. A high bone loss occurs during unloading [2], which may lead to so-called “disuse” osteoporosis [3]. This carries an increased risk of fractures, as seen in e.g. patients with para- and tetraplegia following spine fractures [4]. However, the study of disuse osteoporosis in humans is hampered by the lack of good research models.

Bone turnover can be evaluated using a number of markers of bone formation and resorption. Markers of osteoblastic bone formation include alkaline phosphatase and osteocalcin, whereas procollagen type 1 N-terminal peptide [PINP] is a marker of bone collagen formation [5]. Collagen crosslinks (CTX-I, ICTP) are markers of bone resorption [5]. PINP is a marker of bone collagen, whereas PIIINP is a marker of soft tissue collagen and thus the turnover of muscle collagen. Also markers of calcium metabolism, such as vitamin D metabolites and parathyroid hormone [PTH], are important in assessing pathophysiological differences in the mechanisms of bone loss following physical inactivity. Serum 25-hydroxy-vitamin D (25OHD) reflect vitamin D stores, whereas serum 1,25-dihydroxy-vitamin D (1,25(OH)_2_D) is the circulating active vitamin D that is produced in the kidneys and participate in calcium homeostasis [6].

Hibernating brown bears (*Ursus arctos*) stay inside their winter dens for 5–7 months in Scandinavia [7]; [8] and during this hibernating period they do not eat, drink, defecate, urinate, or have any physical activity. Brown bears are thus highly physically inactive during hibernation, but no signs of disuse osteoporosis have been found in studies of bone structure in bears [9]–[12], i.e. the cortical porosity does not increase**,** which is in contrast to what is observed in immobilised humans [4]. Therefore bears may constitute a model for preventing and treating osteoporosis following unloading, as seen in immobilised patients, as a consequence of a sedentary life style [13], and the bone loss seen in astronauts during prolonged space flights [14].

Little is known about 25OHD levels in bears, as only total 25OHD has been measured previously [15]–[17] with a non-significant decrease being reported during hibernation [15].

It has been shown in humans that differences may exist in the bioavailability and bioactivity of ergocalciferol (vitamin D_2_) and cholecalciferol (vitamin D_3_) [18]. This may be of particular interest in bears, as the diet of bears could result in a different ratio between D_2_ and D_3_ compared to what is typically found in humans, where D_3_ is the predominant form of vitamin D [18].

The aims of our study were to evaluate seasonal differences in bone turnover between active and hibernating brown bears with osteocalcin as a formative marker and ICTP and CTX-I as resorptive markers. Furthermore, we aimed at measuring seasonal variations in 25OHD levels as estimated by total serum 25OHD, serum 25OHD_2_ **,** and serum 25OHD_3._ We also measured the calcitropic hormones serum 1,25(OH)_2_D and serum PTH. Finally, we evaluated connective tissue turnover by measuring serum PIIINP.

## Materials and Methods

### Ethics statement

All animal work has been conducted according to relevant national and international guidelines.

This specific study of bears was approved by the Swedish Ethical Committee on Animal Research, Uppsala (C212/9)**,** and the Swedish Environmental Protection Agency approved the capture of the bears (Dnr 412-7327-09 Nv). All procedures described were in compliance with Swedish laws and regulations.

### Methods

We collected blood samples from 7 previously radio-collared free-ranging two- to three-year-old brown bears (3 females, 4 males) during both hibernation (February 2010) and their active period in the summer (June 2010). The free-ranging bears in our study were monitored throughout the year using GPS collars or VHF transmitters from they were one year old, and we monitored them indirectly before that, because their mothers were monitored in the same way. The bears were immobilized in the den in February and from a helicopter in June by darting with a mixture of tiletamine-zolazepam and medetomidine [19]. Blood was drawn from the jugular vein as described previously [20], centrifuged and kept frozen at −80°C until analysis [20].

This experimental design provides controls for both internal and external effects (body and external temperature, feeding status) in accordance with recommendations by Carey et al. [21].

We performed the following analyses:

1. Osteocalcin (N-MID Osteocalcin), CTX-I (β–Crosslaps), and PTH were analysed in plasma by electrochemilumin**e**scence assays on an automated system (Cobas e601, Roche Diagnostics, Mannheim Germany). Imprecision of the methods was validated over 20 days and coefficients of variation for osteocalcin were 1.0% and 1.1% at levels of 25 and 84 µg/l, respectively, for CTX-I the values were 2.0% and 1.5% at levels of 0.71 and 2.77 µg/l**,** respectively, and for PTH 7.0% and 8.9% at levels of 1.4 and 2.8 pmol/l**,** respectively.
2. ICTP and PIIINP were analysed in serum using radioimmunoassays from Orion Diagnostic**s** (Espoo, Finland). As only small amounts of sample material were available samples were diluted prior to analysis and PIIINP was analyzed by a sequential saturation procedure as described earlier [22]. CV values for PIIINP were 10.2% and 7.1% at levels of 4.9 and 11.7 µg/l, and the corresponding values for ICTP were 8.0% and 9.5% at levels of 7.9 and 22.7 µg/l**,** respectively.
3. 25OHD (incl. 25OHD_3_ and 25OHD_2_) were analysed in serum by isotope dilution liquid chromatography-mass spectrometry (LC-MS/MS) using calibrators traceable to the international standard reference material NIST SRM 972 [23]. Mean CVs for 25OHD_3_ were 8.1% and 9**.**6% at 48 nmol/l and 25 nmol/l, respectively, and for 25OHD_2_ the CV values were 8.5% and 8.0% at levels of 23 and 64 nmol/l, respectively. 1,25(OH)_2_D was analysed by RIA after immunoextraction of the samples (1,25-dihydroxy vitamin D RIA, IDS, Boldon, UK). According to the supplier the method co-determines 1,25(OH)_2_D_2_ with a cross specific**i**ty of 92% compared to 1,25(OH)_2_D_3_. Mean CV values of 6.8% and 9**.**0% were observed at levels of 90 and 220 pmol/l**,** respectively.
4. Serum total calcium was analysed with a routine chemistry analyzer (Cobas c)

### Statistics

We used the mean and standard error of the mean (SEM) as descriptive statistics. The distribution of the variables was tested and found to follow a Gaussian distribution except for 25OHD, 25OHD_2_, 25OHD_3_, and 1,25(OH)_2_D. Despite the fact that these parameters demonstrated a Gaussian distribution after log transformation we chose to compare variables using the Wilcoxon test for paired samples due to the low number of observations. Pearson's correlation coefficient was used to explore correlations. Application of Spearman's rank correlation did not change the results. A p value below 0.05 was considered significant. Borderline significance as indicator of a trend was considered for 0.05<p<0.10.

We performed a power calcification assuming summer 25OHD levels to be 50 nmol/l with a 30% reduction in winter, a standard deviation of 14 nmol/l, a type 1 error of 5% and a type 2 error of 20%. This calculation predicted that 7 animals were necessary in case of paired samples in winter and summer. Due to larger changes in the other variables this was the largest number of animals predicted.

## Results

Table 1 provides baseline data for all analyses performed and compares hibernation with the active summer state. One resorptive marker of bone turnover (ICTP) demonstrated a non-significant trend towards higher values in summer, whereas another marker (CTX-I) showed unchanged levels at the times of sampling. The formative marker osteocalcin was twice as high in summer as in winter, but total serum calcium and PTH did not differ. Total 25OHD tended to be lower in summer than in winter, but the difference was not statistically significant. However, there were significant seasonal shifts in 25OHD_2_ and 25OHD_3_ levels. Serum 25OHD_2_ decreased significantly from winter to summer, whereas serum 25OHD_3_ increased significantly from winter to summer. The values of the active 1,25(OH)_2_D metabolite tended to be higher in summer, but the differences were not statistically significant, because of large standard deviations.

**Table 1:** Baseline characteristics of the biochemical parameters of 7 subadult brown bears in Sweden in 2010 during hibernation in the winter and the active state in the summer and comparisons of hibernation and active state values.

Parameter	Hibernation	Summer	Two-tailed p value (2 p)
ICTP (µg/l)	24.3±2.0	27.2±2.1	0.09
CTX-I (µg/l)	1.7±0.2	2.1±0.2	0.13
Osteocalcin (µg/l)	27.7±2.9	51.2±3.6	0.02
PIIINP (µg/l)	16.4±2.8	67.3±19.9	0.02
25OHD (nmol/l)	63.0±9.1	41.5±3.9	0.09
25OHD_2_ (nmol/l)	54.2±8.3	18.7±1.7	0.02
25OHD_3_ (nmol/l)	8.8±2.1	22.8±4.6	0.03
1,25(OH)_2_D (pmol/l)	88.6±3.3	192.5±59.0	0.07
PTH (pmol/l)	3.9±0.6	4.4±0.7	0.50
Total calcium (mmol/l)	2.39±0.02	2.41±0.04	0.29

Figures are mean±SEM.

Comparisons are made by the Wilcoxon test for paired samples.

Table 2 shows the correlations between the various parameters by season. Total 25OHD levels correlated with 25OHD_3_ levels in summer and 25OHD_2_ levels in winter. In summer, 1,25(OH)_2_D levels correlated with total 25OHD and with 25OHD_3_ levels, whereas this was not the case in winter. In summer PTH levels correlated inversely with serum calcium levels, i.e. PTH decreased with increasing calcium levels, but this was not the case in winter. In winter ICTP tended to correlate inversely with 1,25(OH)_2_D (p = 0.08), whereas this was not the case in summer.

**Table 2:** Pearson's correlation coefficient between parameters of bone turnover and calcitropic hormones of 7 subadult bears in Sweden in 2010 during hibernation in winter and the active state in the summer.

Season	Parameter	ICTP	CTX-I	25OHD	25OHD_2_	25OHD_3_	1,25(OH)_2_D	Osteocalcin	PIIINP	PTH	Total Ca
Summer	ICTP	-	0.65	−0.18	−0.51	0.04	−0.34	0.06	0.25	0.35	−0.48
	CTX-I	-	-	0.22	0.08	0.15	0.01	0.31	−0.02	0.15	−0.21
	25OHD	-	-	-	−0.20	0.93*	0.91*	−0.44	0.38	−0.15	0.25
	25OHD_2_	-	-	-	-	−0.55	−0.25	0.46	−0.52	−0.46	0.48
	25OHD_3_	-	-	-	-	-	0.87*	−0.55	0.52	0.05	0.04
	1,25(OH)_2_D	-	-	-	-	-	-	0.39	0.16	0.003	0.07
	Osteocalcin	-	-	-	-	-	-	-	−0.19	0.39	−0.51
	PIIINP	-	-	-	-	-	-	-	-	0.09	−0.07
	PTH	-	-	-	-	-	-	-	-	-	−0.83*
	Total Ca	-	-	-	-	-	-	-	-	-	-
Winter	ICTP	-	0.48	0.14	0.29	−0.54	−0.78 a	0.41	−0.02	0.54	−0.59
	CTX-I	-	-	−0.02	0.07	−0.34	−0.41	0.09	−0.01	0.78*	0.18
	25OHD	-	-	-	0.97*	0.47	−0.04	−0.39	0.16	−0.06	−0.25
	25OHD_2_	-	-	-	-	0.26	−0.22	−0.30	0.18	0.03	−0.37
	25OHD_3_	-	-	-	-	-	0.68	−0.48	−0.005	−0.37	0.35
	1,25(OH)_2_D	-	-	-	-	-	-	−0.55	−0.56	−0.49	0.27
	Osteocalcin	-	-	-	-	-	-	-	0.63	0.09	0.37
	PIIINP	-	-	-	-	-	-	-	-	-0.22	0.45
	PTH	-	-	-	-	-	-	-	-	-	0.30
	Total Ca	-	-	-	-	-	-	-	-	-	-

a p = 0.08, *Significant correlation, i.e. p<0.05.

## Discussion

In this study we have documented significant differences in 25OHD levels in brown bears between the hibernation period in winter and the active period in summer. This may have significant impact on bone turnover, as the higher levels especially of 25OHD_2_ during hibernation may prevent bone loss.

The precise mechanisms by which hibernating bears prevent disuse bone loss are not known. In American black bears (*Ursus americanus*) an *increased* bone turnover with a maintained, but delayed, coupling between resorption (serum ICTP) and formation (serum osteocalcin and serum PICP) has been reported [15]. Serum levels of PTH were higher during hibernation and especially in the post-hibernation season with a positive correlation between serum PTH and serum osteocalcin. This suggests that the increase in PTH enhances bone turnover and stimulates osteoblastic activity, although other mechanisms involving insulin-like growth factor 1 (IGF-1) and prostaglandin E_2_ (PGE_2_) also may be involved. Furthermore, the rise in PTH may conserve calcium for skeletal recycling by increasing the renal tubular reabsorption of calcium [15]. However, this is the opposite of what is seen in humans, where PTH is suppressed during immobilisation [24]. Moreover in contrast to findings in humans [4] and black bears, studies in grizzly bears (*Ursus arctos horribilis*) have shown a decrease in cortical bone turnover during hibernation, with a balance between formation and resorption that maintain bone structure, porosity, and strength [12]. However, in both bear species trabecular bone mass, structure, and integrity appear to be preserved during hibernation [9]. Until now all studies on bears have been conducted by one group of researchers [9]–[12]; [25] and on other bear species than *Ursus arctos*.

### Vitamin D

Our study showed interesting results for both 25OHD and 1,25(OH)_2_D. Total levels of 25OHD changed little, but this masked considerable changes in 25OHD_2_ and 25OHD_3_. Ergocalciferol (vitamin D_2_) is typically formed in fungi and plants after exposure to sunlight, whereas vitamin D_3_ is formed from cholesterol in the skin after exposure to ultraviolet B (UVB) rays from sunlight. Vitamin D_3_ may also come from the ingestion of cholecalciferol. Our results demonstrated higher 25OHD_3_ levels in summer, suggesting ingestion from food rich in vitamin D_3_ [26] and to some degree dermal production from exposure to sunlight. It is unclear how much sunlight contributes in bears, but other animals are capable of synthesizing vitamin D_3_ despite having fur [27]. The high levels of 25OHD_2_ during winter may indicate mobilisation from pre-hibernation stores accumulated from ingested vitamin D_2_ in food that is stored in fat and metabolised during hibernation. The 25OHD_2_ may originate from a number of food sources. One source of vitamin D may be blueberries that are infested by fungi that produce vitamin D_2_ [26]; [28]; [29]. Other sources of vitamin D_2_ may be plants, such as sun exposed and dried hay [30]; [31], alfalfa hay [32], or rye grass [33]. However, the free-ranging bears in our study were monitored throughout the year using GPS or VHF transmitters, so that we could document that none of the bears resided in cultivated areas or areas with grassland. It is therefore unlikely that vitamin D in our bears stems from these domesticated plants.

Vitamin D_2_ may be less biologically active than vitamin D_3_ [34]. This may explain the very high levels of 25OHD_2_ during hibernation, as these high levels may be necessary to obtain the same biological activity as lower levels of 25OHD_3_ [18]; [34].

The active 1,25(OH)_2_D levels tended to increase from winter to summer, but the change was not statistically significant (p = 0.10). This tendency towards an increase in 1,25(OH)_2_D perhaps reflects higher sun exposure and increased access to animal vitamin D_3_, as indicated by the positive correlation between 25OHD_3_ and 1,25(OH)_2_D during summer. Another explanation could be a higher need for calcium absorption in the intestine and re-absorption in the kidneys during summer to satisfy skeletal needs.

In the study by Donahue et al. [15] the method used for determining 25OHD captured 25OHD_3_, but may not have determined 25OHD_2_ completely, as the manufacturer ALPCO has noted that only 68% of 25OHD_2_ may be determined in comparison with 25OHD_3_. In contrast, our LC-MS method captures both 25OHD_3_ and 25OHD_2_ completely. This may explain why Donahue et al. [15] did not observe changes in total 25OHD levels (i.e. the combination of 25OHD_2_ and 25OHD_3_).

### Serum calcium

In our study serum total calcium remained constant in both seasons and in the same range as in humans (2.20–2.55 mmol/l in humans and around 2.40 mmol/l in bears). This is in contrast with the results of Donahue et al. for American black bears [15] where ionised calcium levels (0.71 during hibernation and 0.96 mmol/l after hibernation) were significantly lower than in humans (around 1.17–1.32 mmol/l). Another study on American black bears found serum calcium levels in the same range as we did, but failed to show any changes between hibernation and summer [35]. Serum calcium levels do not provide exact information about calcium flow through the system, but rather reflect that the different hormones and tissue compartments through various feed back systems that attempt to maintain constant total calcium levels.

### Bone turnover

We found that markers of bone resorption tended to be higher during summer than winter. This is in contrast to the doubling of the formative marker osteocalcin from winter to summer. It thus seems that bone formation may be high in the summer, perhaps reflecting a much higher loading of the bones, whereas in the winter less remodelling is needed to maintain the same bone mineral density. Another explanation could be that the increase in 1,25(OH)_2_D during summer might stimulate osteoblastic osteocalcin production directly in parallel with the observation that exogenous 1,25(OH)_2_D acutely increases serum osteocalcin in humans [36].

The study of American black bears by Donahue et al. [15] showed the opposite of our results, with much higher osteocalcin levels during hibernation than during pre- and post-hibernation. These authors also found a trend towards higher ICTP levels during hibernation than during the active state, which also contrasts with our results. In addition to species differences, another reason for the differences could be that Donahue et al. [15] studied female bears in captivity, several of which had given birth to cubs before the study, whereas we studied subadult male and female bears in the wild.

When interpreting the results regarding osteocalcin, it should be remembered that recent research in humans has shown that osteocalcin is not just a marker of bone turnover, but also a marker of insulin resistance and blood glucose levels, as it is part of the intricate system of pancreatic beta-cell signalling [37]–[39]. Therefore, it is possible that the much higher levels of osteocalcin than we found during summer are related to feeding status with a higher need of insulin for digestion and metabolism during summer. Obviously, significant changes in body composition take place from winter to summer with fat depots that were stored during the summer being mobilised during winter. However, an earlier study of American black bears [40] found no changes in serum insulin levels over time and no correlation between insulin and body weight. In summer, a higher metabolism is needed compared with winter, because of higher physical activity. However, the higher physical activity in summer may also mean that less insulin is needed because of increased insulin sensitivity. Therefore, insulin levels may remain unchanged in response to the complex changes in metabolism.

### Parathyroid hormone (PTH)

In our study PTH levels changed little from winter to summer, in accordance with the constant serum calcium levels. Our results contrast with the results of Donahue et al. [15], who observed an increase during hibernation in black bears. One reason for the absence of any increase in PTH (secondary hyperparathyroidism) during winter may be the presence of sufficient levels of 25OHD metabolites throughout the year may. However, we have no explanation for the observed doubling in serum 1,25(OH)_2_D during summer. Renal 1,25(OH)_2_D production in humans is normally enhanced by PTH, hypocalcaemia, and hypophosphatemia and inhibited by 1,25(OH)_2_D and fibroblast growth factor 23 (FGF23). The latter is a skeletal phosphatonin that increases renal phosphate excretion and acts as a negative regulator of the renal 1-alpha-hydroxylase, which turns 25OHD into 1,25(OH)_2_D [41]. We did not measure these variables in our study, so further studies are necessary.

Donahue et al. [15] presented no data on 1,25(OH)_2_D, thus no direct comparison with our findings were possible.

### Correlations

In summer, 1,25(OH)_2_D levels correlated with 25OHD_3_ levels, whereas 25OHD_2_ levels correlated with total levels in the winter, underlining the importance of stored vitamin D_2_ for the winter. This may be related to later-summer/fall ingestion of fungi, possibly from blueberries contaminated with fungi [29] or other potential vitamin D_2_ sources, as outlined above.

In summer, there was a clear inverse correlation between PTH and serum total calcium, as is also seen in humans [42]. However, this correlation was absent in winter, indicating that other factors besides PTH may be important at this time, perhaps 1,25(OH)_2_D, which determines long-term serum calcium variations, as the half-life of vitamin D is much longer than PTH.

The seasonal changes in total serum 25OHD and 1,25(OH)_2_D levels did not reach statistical significance. However, the two vitamin D subtypes varied inversely with the highest serum levels of 25OHD_2_ during winter and highest serum 25OHD_3_ levels during summer. The higher proportion of 25OHD_2_ to 25OHD_3_ during winter may compensate for the lesser activity of 25OHD_2_ compared with 25OHD_3_ and result in an unchanged total vitamin D impact on target tissues across seasons. Through local and systemic 1-alpha-hydroxylase activity this might contribute to the steady serum PTH levels which did not display seasonal changes. Our observation that serum ICTP correlated inversely with 1,25(OH)_2_D in winter suggests that sufficient levels of active vitamin D are important during hibernation to suppress bone resorption and thereby prevent bone loss. A correlation between ICTP and 1,25(OH)_2_D was not observed during summer.

### Connective tissue and muscles

PIIINP is a marker of the synthesis of connective tissue (collagen type III) and thus also to some degree of muscle status. PIIINP showed much higher levels during summer, possibly reflecting higher connective tissue synthesis during the physical activity in the summer than in the winter, when a lower turnover is needed to conserve the same amount of connective tissue.

### Limitations

A number of limitations exist for the current study relating to the small sample size, potential error in assays because of limited species specificity, lack of dietary calcium measurements, and lack of measurements of biochemical parameters such as FGF23, phosphate, and vitamin D receptor subtypes. However, our study design, where each bear served as its own control is a powerful design controlling for most potential confounding variables. Had we used captive bears, most biochemical limitations could have been avoided but at the cost of introducing other limitations because of diet, decreased physical activity and disturbances.

### Conclusions

We postulate that a carefully coordinated steady state of bone and connective tissue is maintained in brown bears by increased regeneration and turnover during the high activity of the summer and a lower turnover in winter, when lower physical load requires less connective tissue and thus muscle and bone formation. Because hibernating brown bears do not develop disuse osteoporosis despite extensive physical inactivity we suggest that they may serve as a model for the prevention of this disease. The coordinated steady state of muscle and bone in bears should be the subject for future research.

## References

[1] Frost H (1987). Bone “mass” and the “mechanostat”: a proposal.. Anat Rec.

[2] Biering Sorensen F, Bohr H, Schaadt O (1990). Longitudinal study of bone mineral content in the lumbar spine, the forearm and the lower extremities after spinal cord injury.. Eur J Clin Invest.

[3] Abramson A, Delagi E (1961). Influence of weight-bearing and muscle contraction on disuse osteoporosis.. Arch Phys Med Rehab.

[4] Vestergaard P, Krogh K, Rejnmark L, Mosekilde L (1998). Fracture rates and risk factors for fractures in patients with spinal cord injury.. Spinal Cord.

[5] Camacho PM, Lopez NA (2008). Use of biochemical markers of bone turnover in the management of postmenopausal osteoporosis.. Clin Chem Lab Med.

[6] Holick M (2003). Vitamin D: A millenium perspective.. J Cell Biochem.

[7] Manchi S, Swenson J (2005). Denning behaviour of Scandinavian brown bears Ursus arctos.. Wildlife Biology.

[8] Friebe A, Swenson J, Sandegren F (2001). Denning chronology of female brown bears in central Sweden.. Ursus.

[9] McGee-Lawrence ME, Wojda SJ, Barlow LN, Drummer TD, Bunnell K et al (2009). Six months of disuse during hibernation does not increase intracortical porosity or decrease cortical bone geometry, strength, or mineralization in black bear (Ursus americanus) femurs.. J Biomech.

[10] McGee-Lawrence ME, Carey HV, Donahue SW (2008). Mammalian hibernation as a model of disuse osteoporosis: the effects of physical inactivity on bone metabolism, structure, and strength.. Am J Physiol Regul Integr Comp Physiol.

[11] McGee ME, Miller DL, Auger J, Black HL, Donehue SW (2007). Black bear femoral geometry and cortical porosity are not adversely affected by ageing despite annual periods of disuse (hibernation).. J Anat.

[12] McGee ME, Maki AJ, Johnson SE, Nielson OL, Robbins CT et al (2008). Decreased bone turnover with balanced resorption and formation prevent cortical bone loss during disuse (hibernation) in grizzly bears (Ursus arctos horribilis).. Bone.

[13] McGraw RL, Riggs JE (1994). Osteoporosis, sedentary lifestyle, and increasing hip fractures: pathogenic relationship or differential survival bias.. Calcif Tissue Int.

[14] Schneider VS, LeBlanc A, Huntoon CL (1993). Prevention of space flight induced soft tissue calcification and disuse osteoporosis.. Acta Astronaut.

[15] Donahue SW, Galley SA, Vaughan MR, Patterson-Buckendahl P, Demers LM et al (2006). Parathyroid hormone may maintain bone formation in hibernating black bears (Ursus americanus) to prevent disuse osteoporosis.. J Exp Biol 209 (Pt.

[16] Lin RC, Engeli E, Prowten AW, Erb HN, Ducharme NG et al (2005). Antebrachial fractures in four captive polar bears (Ursus maritimus).. Vet Surg.

[17] Crissey S, Ange K, Slifka K, Bowen P, Stacewicz-Sapuntzakis M et al (2001). Serum concentrations of vitamin D metabolites, vitamins A and E, and carotenoids in six canid and four ursid species at four zoos.. Comp Biochem Physiol A Mol Integr Physiol.

[18] Leventis P, Kiely PDW (2009). The tolerability and biochemical effects of high-dose bolus vitamin D2 and D3 supplementation in patients with vitamin D insufficiency.. Scand J Rheumatol.

[19] Kreeger T, Arnemo J (2007). International Wildlife Veterinary Services,2007.

[20] Fröbert O, Christensen K, Fahlman A, Brunberg S, Josefsson J et al (2010). Platelet function in brown bear (Ursus arctos) compared to man.. Thromb J.

[21] Carey HV, Andrews MT, Martin SL (2003). Mammalian hibernation: cellular and molecular responses to depressed metabolism and low temperature.. Physiol Rev.

[22] Søndergaard K, Heickendorff L, Risteli L, Risteli J, Zachariae H et al (1997). Increased levels of type I and III collagen and hyaluronan in scleroderma skin.. Br J Dermatol.

[23] Højskov CS, Heickendorff L, Møller HJ (2010). High-throughput liquid-liquid extraction and LCMSMS assay for determination of circulating 25(OH) vitamin D3 and D2 in the routine clinical laboratory.. Clin Chim Acta.

[24] Mechanick J, Pomerantz F, Flanagan S, Stein A, Gordon W et al (1997). Parathyroid hormone suppression in spinal cord injury patients is associated with the degree of neurologic impairment and not the level of injury.. Arch Phys Med Rehabil.

[25] McGee-Lawrence ME, Wojda SJ, Barlow LN, Drummer TD, Castillo AB, Kennedy O, Condon KW, Auger J, Black HL, Nelson OL, Robbins CT, Donahue SW (2009). Grizzly bears (Ursus arctos horribilis) and black bears (Ursus americanus) prevent trabecular bone loss during disuse (hibernation).. Bone.

[26] Dahle B, Sørensen O, Wedul E, Swenson J, Sandegren F (1998). The diet of brown bears Ursus arctos in central Scandinavia: effect of access to free-ranging domestic sheep ovis aries.. Wildlife Biology.

[27] Hymøller L, Jensen SK (2010). Vitamin D(3) synthesis in the entire skin surface of dairy cows despite hair coverage.. J Dairy Sci.

[28] Persson I, Wikan S, Swenson J, Mysterud I (2001). The diet of the brown bear Ursus arctosin the Pasvik Valley, northeastern Norway.. Wildlife Biology.

[29] Fan L, Forney CF, Song J, Doucette C, Jordan MA et al (2008). Effect of hot water treatments on quality of highbush blueberries.. J Food Sci.

[30] Newlander J, Riddell W (1952). Rate of Vitamin D Formation in Hay.. J Anim Sci.

[31] Harcourt-Brown F (2002). Textbook of rabbit medicine.. Elsevier, 2002.

[32] Horst R, Reinhardt T, Russell J, Napoli J (1984). The Isolation and identification of Vitamin D2 and Vitamin D3 from Medicago sativa (Alfalfa Plant).. Archives of Biochemistry and Biophysics.

[33] Patterson G, Nes W, Champaign I (1999). Physiology and biochemistry of sterols..

[34] Heaney RP, Recker RR, Grote J, Horst RL, Armas LAG (2011). Vitamin D(3) is more potent than vitamin D(2) in humans.. J Clin Endocrinol Metab.

[35] Floyd T, Nelson RA, Wynne GF (1990). Calcium and bone metabolic homeostasis in active and denning black bears (Ursus americanus)..

[36] Nielsen HK, Brixen K, Kassem M, Mosekilde L (1991). Acute effect of 1,25-dihydroxyvitamin D3, prednisone, and 1,25-dihydroxyvitamin D3 plus prednisone on serum osteocalcin in normal individuals.. J Bone Miner Res.

[37] Lee N, Sowa H, Hinoi E, Ferron M, Ahn J et al (2007). Endocrine regulation of energy metabolism by the skeleton.. Cell.

[38] Ferron M, Hinoi E, Karsenty G, Ducy P (2008). Osteocalcin differentially regulates beta cell and adipocyte gene expression and affects the development of metabolic diseases in wild-type mice.. PNAS.

[39] Confavreux CB, Levine RL, Karsenty G (2009). A paradigm of integrative physiology, the crosstalk between bone and energy metabolisms.. Mol Cell Endocrinol.

[40] Herminghuysen D, Vaughan M, Pace RM, Bagby G, Cook CB (1995). Measurement and seasonal variations of black bear adipose lipoprotein lipase activity.. Physiol Behav.

[41] Imel EA, DiMeglio LA, Hui SL, Carpenter TO, Econs MJ (2010). Treatment of X-linked hypophosphatemia with calcitriol and phosphate increases circulating fibroblast growth factor 23 concentrations.. J Clin Endocrinol Metab.

[42] Schwarz P, Sorensen H, Transbol I (1994). Inter-relations between the calcium set-points of Parfitt and Brown in primary hyperparathyroidism: a sequential citrate and calcium clamp study.. Eur J Clin Invest.

